# The effect of paternal age on intracytoplasmic sperm injection outcome in unexplained infertility

**DOI:** 10.1080/2090598X.2021.1955553

**Published:** 2021-07-26

**Authors:** Haitham Elbardisi, Mohamed Arafa, Neha Singh, Bridget Betts, Ashok Agrawal, Ralf Henkel, Alia A. Al-Hadi, Hasan Burjaq, Alia Alattar, Kareim Khalafalla, Ahmad Majzoub

**Affiliations:** a Urology Department, Hamad Medical Corporation, Doha, Qatar; b Weill Cornell Medicine-Qatar, Education City, Qatar; c Andrology Department, Cairo University, Cairo, Egypt; d American Center for Reproductive Medicine, Cleveland Clinic, Cleveland, OH, USA; e Obstetric & Genecology department King George’s Medical University, Lucknow, Uttar Pradesh, India; f School of Pharmacy, University of Mississippi, Mississippi, USA; g Department of Metabolism, Digestion and Reproduction, Imperial College London, LondonUK; h Department of Medical Bioscience, University of Western Cape, Bellville, South Africa; i Department of Reproductive Medicine, Hamad Medical Corporation, Doha, Qatar

**Keywords:** DNA fragmentation index, oxidation–reduction potential, paternal age, pregnancy, semen, unexplained infertility

## Abstract

**Objective:**

: To examine the effect of paternal age on intracytoplasmic sperm injection (ICSI) outcomes in unexplained infertility

**Subjects and Methods:**

: This retrospective study, done at the Hamad Medical Corporation, Doha, Qatar screened infertile couples who underwent ICSI between 2014 and 2019 for the inclusion and exclusion criteria defining ‘unexplained infertility’. Couples recruited were allocated into two groups: Group A (paternal age <35 years) and Group B (paternal age ≥35 years). Baseline characteristics, investigations including semen and advanced sperm function tests and ICSI records were compared for primary outcomes such as fertilisation, cleavage, clinical pregnancy, miscarriage and live birth; and secondary outcomes such as semen parameters and advanced sperm functions (DNA fragmentation index and oxidation reduction potential).

**Results:**

: We found that final pregnancy outcomes including clinical pregnancy rate (*P* = 0.231), live-birth rate (*P* = 0.143), and miscarriage rates (*P* = 0.466) were not significantly different between the two age groups. Normal fertilisation (*P* = 0.01) and cleavage rate after ICSI (*P* = 0.001) were statistically significant when the age groups were compared. Also, normal sperm morphology was found to be significantly different (*P* = 0.041).

**Conclusions:**

: Advanced paternal age affects sperm morphology, fertilisation and embryo cleavage in ICSI but does not appear to affect clinical pregnancy, miscarriage or live-birth rates. ICSI appears to be a valid fertility treatment option in advancing paternal age.

## Introduction

Infertility is a widely studied area of focus in current scientific literature. Infertility occurs when a couple cannot become pregnant after 1 year of attempting to conceive and can be attributed to a multitude of factors from either partner [1]. Unexplained infertility, in which both partners present with normal reproductive parameters, comprises 15–30% of all infertility cases [2]. Couples with unexplained infertility are common candidates for intracytoplasmic sperm injection (ICSI) cycles, an assisted reproductive technology (ART) procedure.

Socioeconomic transition in the past decades has resulted in many couples deciding to postpone pregnancy. Partners elect to get married later in life, use options for controlling fertility, and acknowledge ART procedures have become more advanced and accessible [3]. More couples postpone parenthood prioritising career, education, financial security, and social trends [4]. With increased paternal age, fecundity and natural conception deteriorate [5]. Regardless of the reasoning behind decisions to delay parenthood, the aged couples must be properly counselled by well-informed practitioners as to the predicted ART outcomes with regards to their sexual maturity.

Extensive research has demonstrated that advanced female age leads to decreased quality of oocytes and higher risk pregnancies overall. In contrast, male gametogenesis continues far into adulthood, which theoretically allows fathers to parent children at much older ages [6]. Current literature suggests that sperm quality may deteriorate with advanced age in parameters including decrease in semen volume [7], decrease in sperm motility, weakening of DNA integrity [8], and lower fertilisation potential rate [9,10].

**Figure 1.**
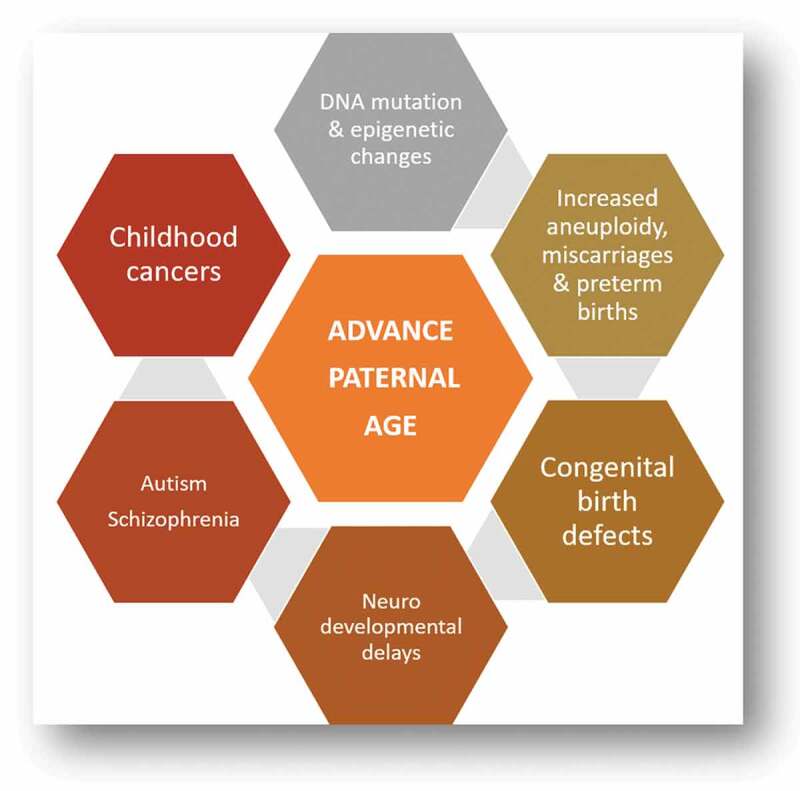
Association of advance paternal age on male reproductive outcomes and offspring’s physical and mental health

It has been proven that advanced paternal age can increase the occurrence of aneuploidies and chromosomal abnormalities, which is potentially linked to the decline in DNA integrity and could be detrimental to pregnancy outcome, in both natural and assisted conception, as shown in Figure 1 [11,12]. That is why, the effect on ICSI outcome is an area of interest for researcher in this field. Many of these studies incorporated the ovum donor model in the setting of severe female factor infertility; however, they might have other factors affecting fertility and ICSI outcomes. Previous studies have not focussed on males and females that present with normal investigations (unexplained infertility), which is why we have selected to study this demographic. Dain et al. [13] in a systematic review concluded that most studies evaluating semen parameter changes with advancing paternal age gave contradictory conclusions yielding discrepancies.

The aims of the present study were to investigate the effects of advanced male age on both basic and advanced semen tests and its impact on the reproductive outcomes with ICSI cycles including fertilisation, cleavage, clinical pregnancy, mischarge and life-birth rate in a setting of unexplained infertility.

## Subjects and methods

### Study population and design

This retrospective study screened all couples enrolling for fertility treatment at Hamad Medical Corporation who underwent ICSI in our centre, a tertiary Centre in Qatar between 1 January 2014 and 1 June 2019. A waiver of informed consent of all patients was taken after due ethical clearance from the Institutional Review Board (IRB No. MRC-01-19-348).

All couples were thoroughly screened for the inclusion and exclusion criteria to define ‘unexplained infertility’ (Table 1).

**Table 1.** Inclusion and exclusion criteria used to screen the study population

Inclusion criteria	Exclusion criteria
At least 1 year of infertility	H/O chemotherapy, radiotherapy, drug addiction or occupational exposure
Females with age <36 years Normal ovarian reserve (AMH ≥15.7 pmol/L) Normal transvaginal US Patent fallopian tube	Wife age ≥36 years Decrease ovarian reserve Presence of hydrosalpinx, endometriosis, PCOD, thin endometrium (<7 mm) or any gynaecological problem
Males with normal semen analysis (WHO fifth edition, 2010) done on two separate occasions.	Abnormal semen report
ICSI cycles with fresh ejaculated sperm	ICSI cycle with frozen or surgically retrieved sperms

PCOD: polycystic ovary disease.

The following data were extracted form medical records of couples undergoing ICSI: age, duration and cause of infertility, medical history, and physical examination. Female’s ultrasonography (US) findings with basal antral follicle count (AFC), hormone profile, tubal status and report of husband’s semen analysis along with details of their ICSI cycles were reviewed.

### Study population

Patients were then allocated into two groups according to the paternal age: younger age group (Group A, paternal age <35 years) and older age group (Group B, paternal age ≥35 years). The groups were them compared for the various primary and secondary outcomes (Table 2).

**Table 2.** Primary and secondary outcomes measured in the study

Primary outcomes
Normal fertilisation = No. of 2 PN fertilized ova/injected eggs
CPR = No. of sac or embryonic pole with or without pulsation/No. of embryo transferred
MR = No. of pregnancy failed to progress beyond 20 weeks/total no. of pregnancy
LBR = No. of full-term delivery/No. of embryo transfer
Secondary outcomes
Semen parameters (volume, sperm concentration, motility, morphology)
SDF
ORP

No.: number outcomes. All primary outcomes (fertilisation rate, CPR, MR, LBR) were defined in accordance with the standard definition by the Vienna Consensus, 2019. Semen parameters according to WHO fifth edition (2010).

### Semen analysis and sperm function test

According to standard laboratory protocol, all male partners were advised 2–3-days abstinence from sexual intercourse and to avoid lubricant before giving semen samples, which were collected in a sterile wide mouth jar by masturbation in a separate room adjacent to the laboratory and liquefied at 37°C for minimum 20 min before examination by a trained andrologist. Physical characteristics of semen like volume (≥1.5 mL), colour (grey), pH (≥7.2) and viscosity were noted. A haemocytometer was used for sperm concentration and air-dried smears were stained with Diff-Quik for morphological assessment of sperm. The percentage of motile (total, progressive and non-progressive) and immotile sperm was scored manually according to WHO fifth edition [14].

Sperm DNA fragmentation (SDF) was assessed by means of the sperm chromatic dispersion test (SCD) by causing acid denaturation and lysis of sperm nuclear protein using the Halosperm G2 Test kit (Halotech, Madrid, Spain) creating a ‘halo’ around the sperm head observed under a microscope at ×400. An upper threshold limit of 30% SDF was used as a cut-off [15].

We determined seminal oxidative stress by means of measuring the oxidation–reduction potential (ORP) with the MiOXSYS system, a galvanostatic-based analyser (Aytu BioScience, Englewood, CO, USA). The disposable sensor was loaded with 30-µL prewashed liquefied semen. When the reference electrode was filled with the sample, the test began because the electrochemical circuit was established and 4 min later, raw ORP was measured in milli Volts (mV), which was then adjusted to sperm concentration. An ORP of >1.34 mV/10^6^ spermatozoa/mL represented high oxidative stress [16].

### Hormone assay

Hormonal investigation included serum anti-Müllerian hormone (AMH; >15.7pmol/L), FSH (1–19 IU/L), LH (1–9 IU/L), oestradiol (E2; 10–60 pg/mL), total testosterone (220–1000 ng/dL), and prolactin (73–407 mIU/L). All blood samples were withdrawn early in the morning between 07:00 and 09:00 hours in the endocrine laboratory of our centre, and measured by chemiluminescence assays, Architect i1000SR® (Abbott Systems, Abbott Park, IL, USA).

### Control ovarian stimulation and embryo transfer

A standard long agonist protocol was followed for ovarian stimulation in all patients. GnRH agonist Gonapeptyl Depot 3.75 mg (Ferring Pharmaceuticals Ltd., West Drayton, UK) was given intramuscular in the mid-luteal phase for pituitary down-regulation. Ovarian stimulation was started with gonadotrophin recombinant FSH (Merck Serono, Darmstadt, Germany) after confirming down-regulation after 14 days by serum E2 < 50 pg/mL, small follicle 4–6 mm, and thin endometrium (<5 mm). The initial gonadotrophin dose was based on patients’ age, body mass index (BMI), AFC, and previous response. Follicular growth was monitored by serial US and serum E2 levels. Once at least two follicles reached 18 mm mean diameter, human chorionic gonadotrophin (hCG) trigger 10,000 IU (IBSA, Pambio-Noranco, Lugano, Switzerland) was given subcutaneously and 34–36 h later ovum retrieval was done transvaginally under US guidance.

Partners gave a fresh semen sample on the day of ovum retrieval, which was processed after liquefaction using a density gradient method according to standard laboratory protocols for ICSI. The oocyte cumulus complex was washed, denuded with strippers and incubated for 2–3 h and all matured MII oocytes were inseminated with husband’s sperm via ICSI and cultured. Fertilisation was confirmed 16–18 h later by presence of two pronuclei. At 72 h after ovum retrieval, all embryos were morphologically graded and two to three good quality cleaved embryos (≥8 regular blastomere with no fragmentation) were transferred via soft catheter in a sterile room under transabdominal US guidance with a semi-filled bladder for optimum visualisation of uterus by an experienced operator. Any difficult embryo transfer was excluded from the study. The extra embryos were frozen.

Serum β-hCG was assessed 14 days after the embryo transfer and if positive, the patient was called for confirmation US 2–3 weeks later. Presence of gestational sac or embryonic pole with or without cardiac activity confirmed clinical pregnancy.

### Statistical analysis

All categorical data are presented with frequency (%) and continuous variables as median with 95% CI. To identify the normal distribution of the study variables the Shapiro–Wilk test for normality was performed. The chi-square test was applied to compare ICSI outcomes between age groups while the Mann–Whitney test was used to compare continuous variables. Statistical analysis was performed using the Statistical Package for the Social Sciences (SPSS®) version 20 (IBM Corp., Armonk, NY, USA). A *P* < 0.05 was considered statistically significant.

## Results

A total of 269 couples were included in the final data set. The study population characteristics are represented in Tables 3 and 4. The median (95% CI) male age was 34.0 (34.028–35.240) years. All hormonal levels and semen parameters were within normal range (Table 3). The total clinical pregnancy rate (CPR) was 51.7%, the miscarriage rate (MR) was 9.5%, and the live-birth rate (LBR) was 37.2% (Table 4).

**Table 3.** Characteristic of the study population and baseline investigations

Variable	Valid, *n*	Median (95% CI)
Clinical Parameters		
Age, years	269	34 (34.028–35.240)
Type of infertility	Unexplained	Unexplained
Years of infertility	>1	>1
E2, pg/mL	124	94 (93.3378–111.022)
FSH, IU/L	118	2.95 (3.5607–4.7566)
LH, IU/L	136	3.45 (3.656–4.302)
Testosterone, ng/dL	154	15.59 (16.16–18.96)
Semen volume, mL	266	2 (1.956–2.403)
Sperm concentration, million/mL	269	54 (55.1088–63.6236)
Right testis size, mL^2^	85	9.8 (9.2516–11.3323)
Left testis size, mL^2^	86	9 (8.9080–10.8315)
Total motility, %	269	55 (54.191–56.626)
Progressive motility, %	269	32 (23.2404–28.5589)
Normal morphology, %	269	15 (17.046–20.248)
SDF, %	82	19 (18.637–24.027)
ORP, mV/10^6^/mL	38	1.42 (1.265–2.157)

**Table 4.** Characteristics of the study population: ICSI outcomes

Variable	Valid, *n*	*N* (%) or median (95% CI)
ICSI outcome	269	
Normal fertilisation (2 PN)	269	7 (7.191–8.229)
Cleaved ICSI	269	7 (6.854–7.890)
Positive (clinical pregnancy)	263	136 (51.7)
Miscarriage	263	25 (9.5)
Live birth	263	98 (37.2)

The number of patients in the younger age group was 144 patients while in the older age group was 125. When comparing the two groups, normal fertilisation was negatively affected by increased paternal age (*P*= 0.01). The number of cleaved embryos was significantly different between the groups (*P*= 0.001). The CPR, although higher in Group A (paternal age <35 years) than in Group B (paternal age ≥35 years) was overall not affected by paternal age (*P*= 0.231). The MR and LBR were not different between the groups (Table 5).

**Table 5.** Comparison of ICSI outcomes between Group A (paternal age <35 years) vs Group B (paternal age ≥35 years)

ICSI outcome	Group A Age <35 years	Group B Age ≥35 years	*P*
Normal fertilisation (2 PN), median (95% CI)	8 (7.584–9.027)	6 (6.191–7.725)	0.01
Cleaved ICSI, median (95% CI)	8 (7.425–8.853)	6 (5.654–7.169)	0.001
Positive (clinical pregnancy), *n* (%)	71 (52.2)	65 (47.8)	0.231
Miscarriage, *n* (%)	13 (9)	12 (10.1)	0.466
Live birth, *n* (%)	49 (34)	49 (41.2)	0.143

There was no significant difference in semen parameters between the two groups except for normal sperm morphology (*P*= 0.041) as shown in Table 6.

**Table 6.** Comparison of clinical parameters between Group A (paternal age <35 years) vs Group B (paternal age ≥35 years)

Clinical variable, median (95% CI)	Group A Age <35 years	Group B Age ≥35 years	*P*
E2, pg/mL	94.3 (93.447–115.8582)	95 (84.1011–116.3941)	0.792
FSH, IU/L	2.85 (3.2365–4.5179)	3 (3.4773–5.8172)	0.409
LH, IU/L	3.4 (3.5733–4.3639)	3.35 (3.4328–4.6426)	0.851
Testosterone, ng/dL	14.65 (15.62–19.41)	17.01 (15.09–19.38)	0.834
Semen volume, mL	2 (1.8531–2.4845)	2.3 (1.8915–2.5506)	0.643
Sperm concentration, million/mL	52.3 (53.8709–65.9527)	56 (53.4994–66.0166)	0.727
Right testis size, mL^2^	12 (9.6019–12.4415)	8.0903 (7.5728–10.7332)	0.114
Left testis size, mL^2^	9.9 (8.9349–11.4914)	8 (7.7775–11.0)	0.359
Total motility, %	55 (54.15–57.725)	55 (52.928–56.316)	0.535
Progressive motility, %	33 (24.2715–31.7646)	30 (19.4794–27.4617)	0.113
Normal morphology, %	12 (15.199–19.467)	15 (17.59–22.628)	0.041
SDF, %	16 (16.542–22.051)	20 (19.39–31.514)	0.076
ORP, mV/10^6^/mL	1.18 (1.0736–2.4738)	1.5189 (1.1446–2.0853)	0.622

## Discussion

Couples with unexplained infertility commonly undergo ICSI cycles to reach pregnancy. Many studies have established that increasing female age can be detrimental to ICSI success, but no clarity exists on if and when to counsel couples regarding ‘the effect of paternal age’ on reproductive potential and pregnancy outcomes. Studies investigating ‘paternal age’ are limited by heterogeneity in methodology [13]. The present study investigated the effects of advanced male age on both basic and advanced semen tests, and how this can affect outcomes of ICSI cycles in unexplained infertility after ruling out all possible confounding risk, so that ‘Paternal Age’ is the only variable evaluated.

In the present study an age cut-off of 35 years was taken after extensive literature search and considering the study design. Although not enough data are available regarding a paternal age threshold, studies have suggested semen quality deteriorates after the age of 35 years [17,18]. Maximum sperm quality is seen between the ages of 30 and 35 years [19]. Also, as we did not have the SDF reports of all our patients, we wanted to include only semen sample with lesser SDF. So, we decided to take a lower cut-off value of paternal age because SDF in semen is shown to increase with advancing age mainly after the age of 35–40 years [20]. Raising the male age threshold further would have also resulted in increasing the overall female partner’s age, thus biasing our study results.

In accordance with the internationally accepted benchmark for ICSI outcome [21], the total CPR in our study group was 51.7% (benchmark >35%). The overall MR was 9.5% (benchmark <25%), while the LBR in the present study population reached 37.2% (benchmark 20–30%).

While analysing semen characteristics between the two groups investigated, we found that sperm morphology was the only parameter differing significantly (Group A 12.0% vs Group B 15.0%, *P*= 0.041). The finding resonated with various previous studies in which morphology declines with increasing paternal age [17,22,23]. However, in all these studies sperm motility and volume decreased with advancing paternal age. According to Stone et al. [17] there is an age-based decline in semen parameters with sperm count decreasing after 35 years, concentration by 40 years, motility by 43 years, and lastly volume after 45 years of age. Similarly, Levitas et al. [19] reported all semen parameters to decline with age so that men in the age group of 30–35 years have maximum sperm quality. Sloter et al. [24] quantified the decline of sperm motion kinetics with advancing age. Therefore, while numerous studies reported decreasing semen quality with age, we did not find any correlations except for morphology. This may be due to the fact that our present study included males with normal semen analysis while most of the previous studies were conducted on sub-fertile or infertile men, known to have abnormal semen quality [25]. Also, in many of these studies, the men were advised to have a longer duration of abstinence compared to the 3–5 days in our present study that might have affected their results.

Furthermore, it was found in our present study that there is no significant effect of ageing on advanced semen parameters such as SDF and ORP, in accord with some other studies [18,26]. Conversely, many previous studies have reported a positive correlation of SDF and ORP with increased paternal age [16,27]. A possible explanation could be that both our present study groups included men with normal semen parameters, hence were expected to have a low level of oxidative stress, which is the main cause for elevated ORP and SDF. Furthermore, due to the retrospective nature of our present study there were some patients with missing ORP and SDF testing, which might have affected our results.

Our present study demonstrated that the average number of eggs fertilised (*P*= 0.01) and embryo cleavage (*P*= 0.001) were significantly higher in the younger cohort (8.0 vs 6.0). Our present findings mirrored those of a study by Aboulghar et al. [28], which evaluated 15,657 ICSI cycles to establish a significantly higher fertilisation rate in the age group <50 years (*P*< 0.001; odds ratio [OR] 1.36, 95% CI 1.19–1.55) with the fertilisation rate dropping by 0.3%/year without significantly decreasing the pregnancy rate (36.6% vs 37.9%; OR 1.06, 95% CI 0.72–1.55). Similarly, Cito et al. [10] scrutinised 278 ICSI donor cycles in paternal age <45 and >45 years to find a negative association of fertilisation rate with increased paternal age (80.0% vs 67.0%, *P*< 0.05). Contradicting our present findings, Beguería et al. [29] after evaluating 4887 donor oocytes ICSI concluded that paternal age does not relate with rate of fertilisation or embryo cleavage but then our present study was done using freshly prepared sperm while that study was done mostly using frozen sperm (75% of the cases). Frozen sperm have been shown to have accelerated SDF, which could be the cause of the difference in outcome [30].

Additionally, in our present study, male age did not seem to significantly affect pregnancy, miscarriage or the LBR after ICSI in agreement with various previous studies [9,10]. Similarly, Beguería et al. [29] in a retrospective analysis of 4887 ICSI cycles using donor eggs highlighted that paternal age does not affect the CPR (OR 0.98, 95% CI 0.94–1.033; *P*= 0.52), MR (OR 1.06, 95% CI 0.94–1.03; *P*= 0.52) or LBR (OR 0.98, 95% CI 0.94–1.03; *P*= 0.52). Nonetheless, there are few studies in which pregnancy outcomes were significantly affected by advancing paternal age [31,32]. Ford [33] also concluded that in males, the chance of conceiving within 12 months decreases by 3%/year. However, in many of these studies female age was not adjusted properly, which might have biased by decreasing the implantation rate.

The fact that advanced paternal age is associated with accumulation of genetic mutations, increased sperm diploidy, epigenetic changes and DNA breaks may have interfered with fertilisation and competency of embryo development and could be a possible explanation for a significant difference in fertilisation and cleavage rates between the study groups [34]. Our present study was well adjusted for all the known variables affecting implantation of the embryo and pregnancy outcomes like embryo quality [35], maternal age [36], endometrial thickness [37] and AMH [38], which might explain why we did not find any a significant difference in the CPR, MR and LBR between the two groups. Moreover, female age, which is a major determinant of fertilisation and implantation, was well controlled in our present study. However, the major limitation of our present study was its retrospective nature due to which we failed to assess some other variables affecting semen parameters like BMI, smoking, and endocrine factors such as thyroid status. Also due to our centre’s protocol, we did not perform blastocyst transfer; hence, we could not see the effect of the paternal genome in extended embryo culture to form the blastocyst.

## Conclusion

Advanced paternal age is associated with increases in abnormal sperm morphology and decreases in normal fertilisation and embryo cleavage. Our present findings demonstrate that paternal age does not ultimately affect final pregnancy outcomes including the CPR, LBR, and MR. Interpretation of our present study suggests ICSI to be a satisfactory option in advanced paternal age. Paternal age is an independent factor in improving ICSI outcomes and thus needing due attention while counselling an infertile couple.

## References

[1] T.j. L, Vitrikas KR. Evaluation and treatment of infertility [published correction appears in Am Fam Physician. Am Fam Physician. 2015;91(5): 308–314. 2015 Sep 15;92 (6):437]. 25822387

[2] Johnson LN, Sasson IE, Sammel MD, et al. Does intracytoplasmic sperm injection improve the fertilization rate and decrease the total fertilization failure rate in couples with well-defined unexplained infertility? A systematic review and meta-analysis. Fertil Steril. 2013;100(3):704–711. 23773312 10.1016/j.fertnstert.2013.04.038

[3] Schmidt L, Sobotka T, J.g. B, et al., ESHRE Reproduction and Society Task Force. Demographic and medical consequences of the postponement of parenthood. Hum Reprod Update. 2012;18(1):29–43. . 21989171 10.1093/humupd/dmr040

[4] Mills M, Rindfuss RR, McDonald P, et al., ESHRE Reproduction and Society Task Force. Why do people postpone parenthood? Reasons and social policy incentives. Hum Reprod Update. 2011;17(6):848–860. . 21652599 10.1093/humupd/dmr026 PMC3529638

[5] Hassan MA, Killick SR. Effect of male age on fertility: evidence for the decline in male fertility with increasing age. Fertil Steril. 2003;79(3):1520–1527. 12801554 10.1016/s0015-0282(03)00366-2

[6] Gallo M, Licata E, Meneghini C, et al. Impact of Paternal Age on Seminal Parameters and Reproductive Outcome of Intracytoplasmic Sperm Injection in Infertile Italian Women. Front Endocrinol (Lausanne). 2019;10:35. Published 2019 Feb 13. . 30814975 10.3389/fendo.2019.00035 PMC6381013

[7] Kumar N, Singh AK, Choudhari AR. Impact of age on semen parameters in male partners of infertile couples in a rural tertiary care center of central India: a cross-sectional study. Int J Reprod Biomed (Yazd). 2017;15(8):497–502. PMC5653911 29082368

[8] Hammiche F, Laven JS, Boxmeer JC, et al. Sperm quality decline among men below 60 years of age undergoing IVF or ICSI treatment. J Androl. 2011;32(1):70–76. 20467050 10.2164/jandrol.109.009647

[9] Bartolacci A, Pagliardini L, Makieva S, et al. Abnormal sperm concentration and motility as well as advanced paternal age compromise early embryonic development but not pregnancy outcomes: a retrospective study of 1266 ICSI cycles. J Assist Reprod Genet. 2018;35(10):1897–1903. 29995229 10.1007/s10815-018-1256-8 PMC6150884

[10] Cito G, Coccia ME, Picone R, et al. Impact of advanced paternal age on the intracytoplasmic sperm injection (ICSI) outcomes in donor egg cycles. Transl Androl Urol. 2019;8(1):S22–S30. 31143668 10.21037/tau.2018.12.13 PMC6511697

[11] Brandt JS, Cruz Ithier MA, Rosen T, et al. Advanced paternal age, infertility, and reproductive risks: a review of the literature. Prenat Diagn. 2019;39(2):81–87. 30520056 10.1002/pd.5402

[12] García-Ferreyra J, Luna D, Villegas L, et al. High Aneuploidy Rates Observed in Embryos Derived from Donated Oocytes are Related to Male Aging and High Percentages of Sperm DNA Fragmentation. Clin Med Insights Reprod Health. 2015;9:21–27. Published 2015 Nov 11. . 26604851 10.4137/CMRH.S32769 PMC4642825

[13] Dain L, Auslander R, Dirnfeld M. The effect of paternal age on assisted reproduction outcome. Fertil Steril. 2011;95(1):1–8. 20932518 10.1016/j.fertnstert.2010.08.029

[14] World Health Organization.2010.WHO laboratory manual for the examination and processing of human semen.(5th ed.) .World Health Organization.

[15] Fernández JL, Muriel L, Goyanes V, et al. Simple determination of human sperm DNA fragmentation with an improved sperm chromatin dispersion test. Fertil Steril. 2005;84(4):833–842. 16213830 10.1016/j.fertnstert.2004.11.089

[16] Agarwal A, Bui AD. Oxidation-reduction potential as a new marker for oxidative stress: correlation to male infertility. Investig Clin Urol. 2017;58(6):385–399. 10.4111/icu.2017.58.6.385 PMC5671957 29124237

[17] Stone BA, Alex A, Werlin LB, et al. Age thresholds for changes in semen parameters in men. Fertil Steril. 2013;100(4):952–958. 23809502 10.1016/j.fertnstert.2013.05.046

[18] Brahem S, Mehdi M, Elghezal H, et al. The effects of male aging on semen quality, sperm DNA fragmentation and chromosomal abnormalities in an infertile population. J Assist Reprod Genet. 2011;28(5):425–432. 21287403 10.1007/s10815-011-9537-5 PMC3151353

[19] Levitas E, Lunenfeld E, Weisz N, et al. Relationship between age and semen parameters in men with normal sperm concentration: analysis of 6022 semen samples. Andrologia. 2007;39(2):45–50. 17430422 10.1111/j.1439-0272.2007.00761.x

[20] Vagnini L, Baruffi RL, Mauri AL, et al. The effects of male age on sperm DNA damage in an infertile population. Reprod Biomed Online. 2007;15(5):514–519. 10.1016/S1472-6483(10)60382-3PMID:18028741 18028741

[21] Vienna consensus: report of an expert meeting on the development of ART laboratory performance indicators ESHRE Special Interest Group of Embryology and Alpha Scientists in Reproductive Medicine a, b, * a European Society of Human Reproduction and Embryology, Meerstraat 60, B-1852 Grimbergen, Belgium b ALPHA Scientists in Reproductive Medicine, 19 Mayis Mah. 19 Mayis Cad. Nova Baran Center No:4 34360 Sisli, Istanbul, Turkey.

[22] Molina RI, Martini AC, Tissera A, Olmedo J, Senestrari D, de Cuneo MF, Ruiz RD. Semen quality and aging: analysis of 9.168 samples in Cordoba. Argentina. Arch Esp Urol. 2010;63(3):214–222. English, Spanish. PMID: 20431185. 20431185

[23] Kidd SA, Eskenazi B, Wyrobek AJ. Effects of male age on semen quality and fertility: a review of the literature. Fertil Steril. 2001;75(2):237–248. 11172821 10.1016/s0015-0282(00)01679-4

[24] Sloter E, Schmid TE, Marchetti F, et al. Quantitative effects of male age on sperm motion. Hum Reprod. 2006;21(11):2868–2875. 16793993 10.1093/humrep/del250

[25] Das M, Al-Hathal N, San-Gabriel M, et al. High prevalence of isolated sperm DNA damage in infertile men with advanced paternal age. J Assist Reprod Genet. 2013;30(6):843–848. 23722935 10.1007/s10815-013-0015-0 PMC3696445

[26] Colin A, Barroso G, Gómez-López N, et al. The effect of age on the expression of apoptosis biomarkers in human spermatozoa. Fertil Steril. 2010;94(7):2609–2614. 20542266 10.1016/j.fertnstert.2010.04.043

[27] Schmid TE, Eskenazi B, Baumgartner A, et al. The effects of male age on sperm DNA damage in healthy non-smokers. Hum Reprod. 2007;22(1):180–187. 17053003 10.1093/humrep/del338

[28] Aboulghar M, Mansour R, Al-Inany H, et al. Paternal age and outcome of intracytoplasmic sperm injection. Reprod Biomed Online. 2007;14(5):588–592. 17509198 10.1016/s1472-6483(10)61050-4

[29] Beguería R, García D, Obradors A, et al. Paternal age and assisted reproductive outcomes in ICSI donor oocytes: is there an effect of older fathers? Hum Reprod. 2014 October 10;29(10):2114–2122. . 25073975 10.1093/humrep/deu189 PMC4164148

[30] Gosálvez J, Núñez R, Fernández JL, et al. Dynamics of sperm DNA damage in fresh versus frozen-thawed and gradient processed ejaculates in human donors. Andrologia. 2011;43(6):373–377. 21919930 10.1111/j.1439-0272.2010.01022.x

[31] Chapuis A, Gala A, FerriÃ¨res-Hoa A, et al. Sperm quality and paternal age: effect on blastocyst formation and pregnancy rates. Basic Clin Androl. 2017;27(1):2. . 28127436 10.1186/s12610-016-0045-4 PMC5251225

[32] Klonoff-Cohen HS, Natarajan L. The effect of advancing paternal age on pregnancy and live birth rates in couples undergoing in vitro fertilization or gamete intrafallopian transfer. Am J Obstet Gynecol. 2004;191(2):507–514. 15343228 10.1016/j.ajog.2004.01.035

[33] Ford W. Increasing paternal age is associated with delayed conception in a large population of fertile couples: evidence for declining fecundity in older men. Hum Reprod. 2000;15(8):1703–1708. 10920089 10.1093/humrep/15.8.1703

[34] Sharma R, Agarwal A, Rohra VK, et al. The effect of increased paternal age on sperm quality, reproductive outcome and associated epigenetic risks to offspring. Reprod Biol Endocrinol. 2015;13:35. 25928123 10.1186/s12958-015-0028-x PMC4455614

[35] Cai QF, Wan F, Huang R, et al. Factors predicting the cumulative outcome of IVF/ICSI treatment: a multivariable analysis of 2450 patients. Hum Reprod. 2011 September;26(9):2532–2540. . 21771773 10.1093/humrep/der228

[36] Tan TY, Lau SK, Loh SF, et al. Female ageing and reproductive outcome in assisted reproduction cycles. Singapore Med J. 2014;55(6):305–309. 25017405 10.11622/smedj.2014081 PMC4294057

[37] Yuan X, Saravelos SH, Wang Q, et al. Endometrial thickness as a predictor of pregnancy outcomes in 10787 fresh IVF–ICSI cycles. Reprod Biomed Online. 2016;33(2):197–205. 27238372 10.1016/j.rbmo.2016.05.002

[38] Lin WQ, Yao LN, Zhang DX, et al. The predictive value of anti-Mullerian hormone on embryo quality, blastocyst development, and pregnancy rate following in vitro fertilization-embryo transfer (IVF-ET). J Assist Reprod Genet. 2013;30(5):649–655. 23504440 10.1007/s10815-013-9973-5 PMC3663964

